# Mimicry in Cretaceous Bugs

**DOI:** 10.1016/j.isci.2020.101280

**Published:** 2020-06-16

**Authors:** Erik Tihelka, Michael S. Engel, Diying Huang, Chenyang Cai

**Affiliations:** 1 Department of Animal Science, Hartpury College, Hartpury GL19 3BE, UK; 2 Division of Entomology, Natural History Museum, University of Kansas, Lawrence, KS 66045, USA; 3 Department of Ecology & Evolutionary Biology, University of Kansas, Lawrence, KS 66045-4415, USA; 4 State Key Laboratory of Palaeobiology and Stratigraphy, Nanjing Institute of Geology and Palaeontology, Centre for Excellence in Life and Paleoenvironment, Chinese Academy of Sciences, Nanjing 210008, China; 5 School of Earth Sciences, University of Bristol, Life Sciences Building, Tyndall Avenue, Bristol BS8 1TQ, UK

**Keywords:** Entomology, Evolutionary Biology, Systematics, Phylogenetics, Paleobiology

## Abstract

Mimicry is ubiquitous in nature, yet understanding its origin and evolution is complicated by the scarcity of exceptional fossils that enable behavioral inferences about extinct animals. Here we report bizarre true bugs (Hemiptera) that closely resemble beetles (Coleoptera) from mid-Cretaceous amber. The unusual fossil bugs are described as *Bersta vampirica* gen. et sp. nov. and *Bersta coleopteromorpha* gen. et sp. nov. and are placed into a new family, Berstidae fam. nov. The specialized mouthparts of berstids indicate that they were predaceous on small arthropods. Their striking beetle-like appearance implies that they were either involved in defensive mimicry or mimicked beetles to attack unsuspecting prey. The latter would represent the first case of aggressive mimicry in the invertebrate fossil record. These findings enrich our understanding of the paleoecological associations and extinct behavioral strategies of Mesozoic insects.

## Highlights

• A new family of beetle-mimicking bugs is described from Cretaceous amber
• Berstidae were aggressive or defensive mimics of polyphagan beetles
• They are one of the earliest mimics in the fossil record of insects
• The discovery points at a high ecological complexity in mid-Cretaceous rainforests

## Graphical Abstract

**Figure:**
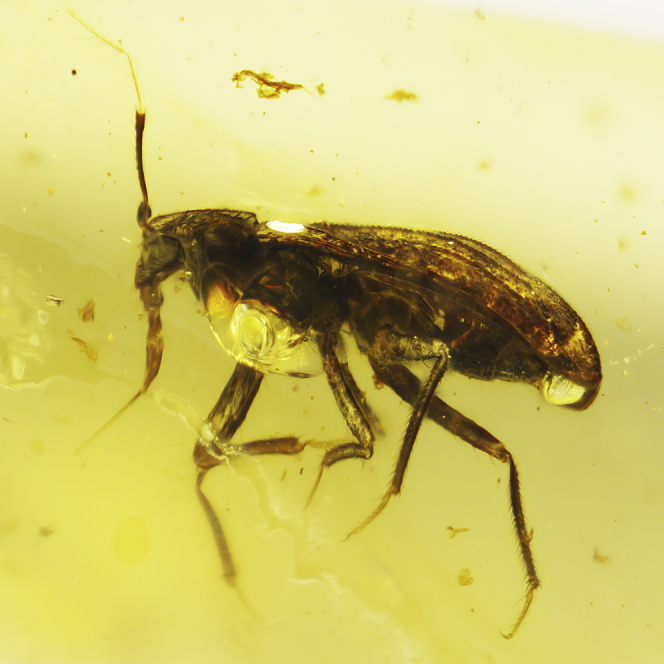

## Introduction

Mimicry, whereby organisms resemble living or inanimate objects, is a keystone concept in evolutionary biology and has received considerable interdisciplinary attention as one of the most vivid illustrations of Darwinian natural selection (Casewell et al., 2017). Mimics deploy a range of elaborate methods, including morphological, physiological, and behavioral adaptations, to avoid recognition by predators, prey, or conspecifics (Pfennig, 2012). Defensive mimicry (e.g., Batesian or Müllerian mimicry) involves species that conceal their true identities to avoid predation. Defensive Batesian mimicry is widespread in some animals such as in some groups of butterflies that resemble toxic species despite being harmless themselves (Barber and Conner, 2007; Pinheiro, 1996). On the other end of the spectrum of mimetic associations in nature, aggressive mimicry (Peckhamian mimicry, Peckham, 1889) involves predators that imitate, often in intricate ways involving visual and chemical deceit, their own prey to avoid being detected (Jamie, 2017). Some of the most striking examples of aggressive mimicry include humpback anglerfish that use their bioluminescent dorsal spine to lure and capture prey, parasitic trematodes that gain entry into their unsuspecting hosts by mimicking their food, and predaceous spiders that, like true “wolfs in sheep's clothing,” mimic harmless species by releasing a cocktail of deceitful chemical cues (Nelson and Jackson, 2012; Pietsch and Grobecker, 1978). However, the fossil record of complex ecological interactions such as mimicry is very sparse, disclosing little about their evolution (Boucot, 1990).

Few reliable examples of mimicry have been described from the fossil record to date (Boucot and Poinar, 2010; Kácha and Petr, 1996; Topper et al., 2015; Vinther et al., 2016), mostly because incomplete preservation of isolated fossils makes paleoecological interpretations difficult (Wang et al., 2012). Among extinct vertebrates, defensive mimicry was hypothesized, among others, in ankylosaurid dinosaurs that may have used their enlarged tail clubs to divert predator attacks from their heads to this well-defended region (Thulborn, 1993). The earliest putative case of aggressive mimicry in vertebrates has been recognized in a Late Jurassic piranha-like pycnodontiform fish that likely used its close resemblance of harmless species to approach prey unnoticed (Kölbl-Ebert et al., 2018). Cases of defensive mimicry in Mesozoic arthropods are rare (Hinkelman, 2020; Vršanský et al., 2018) and no cases of aggressive mimicry have so far been demonstrated in fossil invertebrates (Kácha and Petr, 1996, 1997).

Here we report a new group of beetle-mimicking Cretaceous bugs based on exceptionally preserved fossils in mid-Cretaceous amber from northern Myanmar, one of the most biodiverse insect Lagerstätten from the Mesozoic (Cai et al., 2019; Ross, 2019). These beetle-mimicking bugs are exceptionally rare, only three specimens belonging to two species were found after examining more than 40,000 inclusions. The lifelike fidelity of our fossils reveals morphological characters that identify them as predaceous visual mimics of beetles. Based on their striking beetle-like morphology, the bugs are placed into a new family, Berstidae fam. nov. These extinct Cretaceous bugs belong to the order Cimicomorpha, which includes modern bed bugs and kissing bugs, providing a new insight into the early evolution of this economically important group of insects, as well as a rare window into the ecological complexity of Cretaceous tropical rainforests at the heart of the Cretaceous terrestrial revolution.

## Results

### Systematic Paleontology

Order Hemiptera Linnaeus, 1758, suborder Heteroptera Latreille, 1810.

Infraorder Cimicomorpha Leston, Pendergrast et Southwood, 1954.

Family Berstidae fam. nov.

### Diagnosis

**Figure 1:**
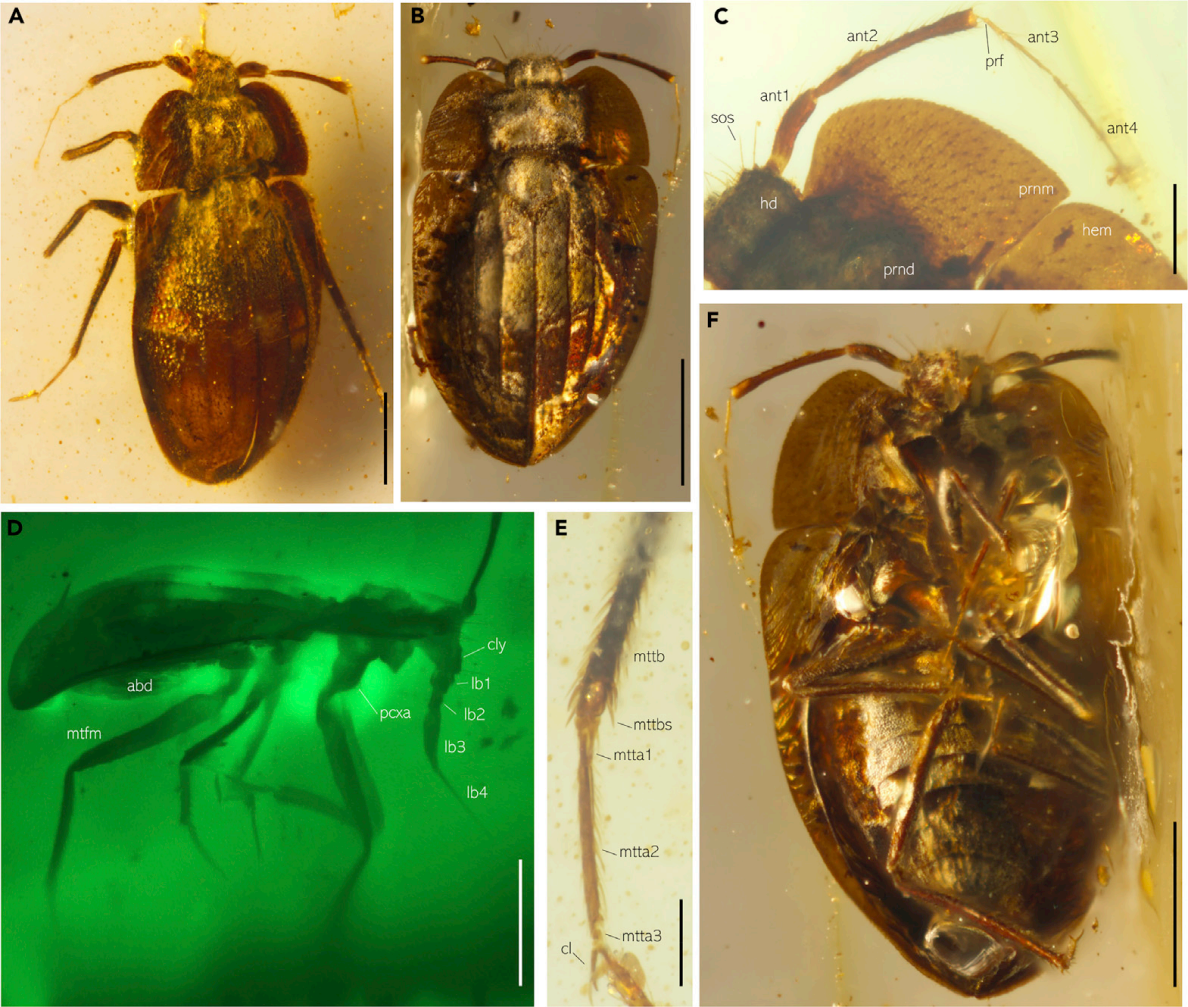
Berstidae fam. nov.: Photographs of Specimens Entombed in Mid-Cretaceous Amber (A) Habitus of *Bersta coleopteromorpha* gen. et sp. nov. under reflected light. (B) Habitus of *Bersta vampirica* gen. et sp. nov. under reflected light. (C) Antenna of *Bersta vampirica* gen. et sp. nov. under reflected light. (D) Lateral view of *Bersta vampirica* gen. et sp. nov. under green fluorescence. (E) Metatarsus of *Bersta coleopteromorpha* gen. et sp. nov. under green fluorescence. (F) Ventral view of *Bersta vampirica* gen. et sp. nov. under reflected light. Scale bars: 500 μm in (A–F) and 200 μm in (C and E). abd, abdomen; ant1–4, antennomere 1–4; cl, claw; cly, clypeus; hd, head; hem, hemelytron; lb 1–4, labial segments 1–4; mtfm, metafemur; mtta1–3, metatarsi 1–3; mttb, metatibia; mttbs, metatibial spine; pcxa, procoxa; prf, preflagelloid; prnd, pronotal disc; prnm, pronotal margin; sos, supraocular seta. See also Figures S1–S3.

Body elongate to subelliptical, glabrous, not covered with layers of setae, coleopteroid (Figure 1). Size rather small, when compared with the remainder of Hemiptera; length ranging from 2.0 to 2.6 mm. Head hypognathous, more or less pentagonal in dorsal view, without a constriction posterior to compound eyes; collar present. Labium four-segmented, tapering apically, inserted on ventral head surface. Antenna with four articles; prepedicellite absent; antennomere II (pedicel) longer than antennomere III (basiflagellomere), antennomeres III and IV (distiflagellomere) filiform, much narrower than preceding antennomeres, with erect setae. Compound eyes well developed, but not surpassing collar posteriorly. Ocelli absent. Thoracic labial groove present; pronotum trapezoidal. Pronotum almost butterfly shaped, with anterior margin sinuate, anterior angles smoothly curved, and posterior angles approximately right angled. Pronotal and hemelytral margins pronouncedly expanded, clearly overlapping sides of thorax and abdominal base. Legs slender and setose, of cursorial type. Prolegs lack any specialist raptorial adaptations. Tarsi trimerous, pretarsal parempodia absent.

### Comparison

Berstids differ from other hemipteran families with a coleopteroid appearance, such as Omaniidae, Schizopteridae, and Tingidae, in having their forewings coriaceous, sclerotized throughout their posterior margins, parallel, and connate (apices not overlapping), each with simple longitudinal veins and lacking a costal fracture. They can be assigned to Cimicomorpha based on the presence of cephalic trichobothria; body not covered with a short hair pile and lacking marginal laminae; head not transversely constricted or divided into two distinct lobes; eyes small; antennae not concealed below head in grooves under compound eyes, longer than head, antennomere 2 longer than antennomere 1; forewings modified into coleopteroid hemelytra with longitudinal carinae; abdominal sterna without trichobothria placed sublaterally or submedially; protibiae not flattened; tarsi three-segmented, claws lacking pulvilli. Within Cimicomorpha, the fully coriaceous, parallel, and non-overlapping hemelytra represent an apomorphy of Berstidae.

### Type Genus

Bersta gen. nov.

Genus *Bersta* gen. nov.

### Type Species

*Bersta vampirica* sp. nov.

### Other Species Included

*Bersta coleopteromorpha* sp. nov.

### Diagnosis

As for the family with the following additional characters, abdominal trichobothria absent, abdominal spiracles on unified sternal plates, each hemelytron with three longitudinal veins, body length ≤ 2.6 mm.

### Etymology

The new generic name is an euphonious combination of letters inspired by “Berstuk” a deity of the Wendic Slavs and Sorbs that, according to myth, inhabited deep forests and had the ability to morph into different animals, and refers to the likely paleoenvironment and paleoecology of the bugs. The name is considered of feminine gender.

### Type Locality and Horizon

Amber mine in the Hukawng Valley, Myitkyina District, Kachin State, Myanmar; Albian/Cenomanian boundary to late Albian (mid-Cretaceous).

### *Bersta coleopteromorpha sp*. *nov.*

Distinguished from *B*. *vampirica* by hemelytral vein 1 not connected to the sutural margin and veins 2 and 3 fading posteriorly, not joining, with the posterior part of vein 1 directed laterally and the posterior part of vein 3 directed medially. Moreover, the pronotum has four clearly defined longitudinal ridges connected to an anterior latitudinal ridge. Unlike its sister species, *B*. *coleopteromorpha* lacks elevated keels to the side of eyes.

### Bersta vampirica *sp*. *nov.*

The species can be differentiated from *B*. *coleopteromorpha* by the hemelytral vein 1 abruptly curving medially in its posterior part and joining the sutural margin and veins 2 and 3 fused posteriorly. Moreover, it differs by the anterior pronotal angles reaching to the posterior part of the antennal insertions, and by pronotum with an indistinct longitudinal and latitudinal ridge forming a raised cross-like structure with four depressions. In addition, it can be distinguished from *B*. *coleopteromorpha* gen. et sp. nov. by the presence of supraocular ridges, each with a single seta.

### Description

For a full description of *Bersta vampirica* sp. nov. and *B*. *coleopteromorpha* sp. nov., please refer to the Supplemental Information. All new taxonomic acts are registered in ZooBank under the publication LSID urn:lsid:zoobank.org:pub:F2D47B4A-0F3A-43AB-814B-3D92A7A9506D.

## Discussion

### Paleoecology of Berstidae

**Figure 2:**
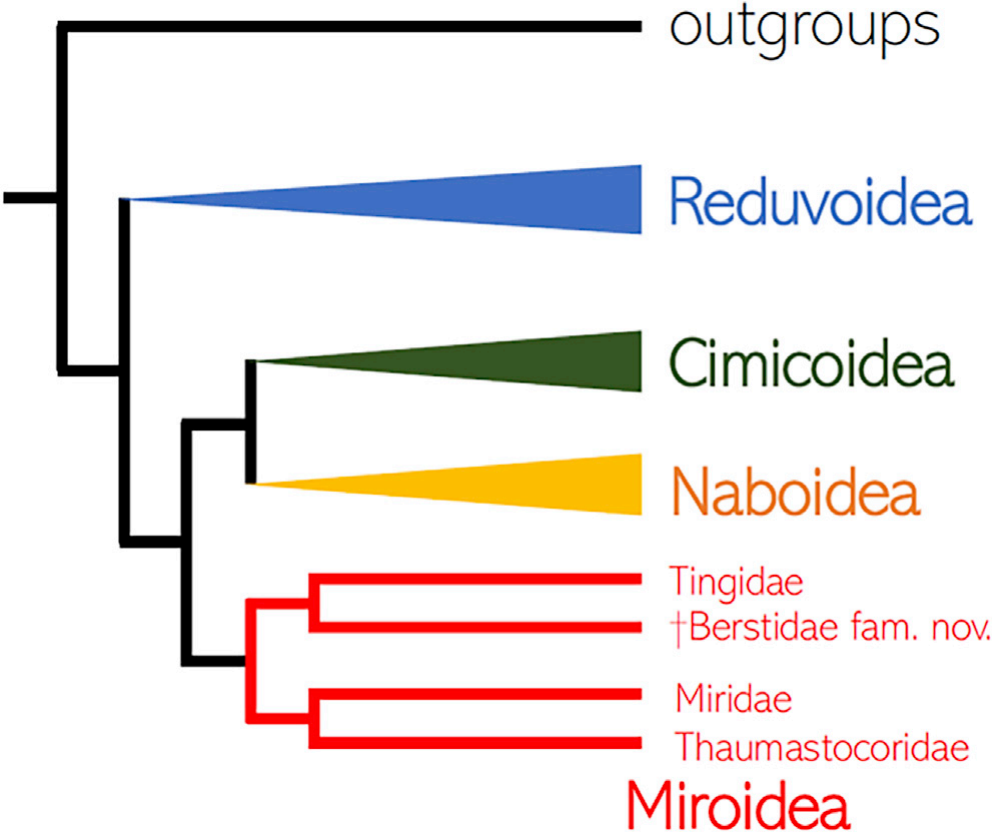
Phylogenetic Hypothesis on the Placement of Berstidae fam. nov. Based on an analysis of mitogenomes and morphological data (Figures S4 and S5).

To determine the systematic position of Berstidae fam. nov., we have conducted a total evidence phylogenetic analysis including morphological data for all extinct and extant families of Cimicomopha. We used 13 mitochondrial protein-coding genes and two mitochondrial rRNA for 9 families to reconstruct the backbone phylogeny of the infraorder, which has so far proven difficult to resolve with morphological data alone (Yang et al., 2018). Berstids have been recovered as a sister group to the family Tingidae based on a maximum parsimony analysis of 81 morphological characters (Figure 2). This affinity is supported by the shared coleopterous structure of the hemelytra. Extant tingids are phytophagous, whereas some members of Miroidea are known to be predaceous (Dolling and Palmer, 1991; Schuh and Slater, 1995).

Even among extant bugs, making behavioral inferences from morphological data alone is difficult (Yao et al., 2014). Nonetheless, morphological evidence from our fossils provides important information that can help approximate the diet of Berstidae. Berstids have a labium that is widest proximally and tapers apically (Figure 1). This condition is present in all predaceous and blood-sucking true bugs, but never occurs in phytophagous species (Cohen, 1990; Yao et al., 2014). The presence of filiform antennae with the apical two antennomeres thinner than the second antennomere appears to be associated with predaceous and hematophagous lifestyles (Yao et al., 2014) and living in dead plant material (Jung and Lee, 2012), although there understandably are exceptions. The presence of this antennal morphotype in Cretaceous amber berstids would be consistent with the notion that pre-Paleogene cimicomorphs lived in dead plant matter (Jung et al., 2010; Jung and Lee, 2012).

A predatory, as opposed to a hematophagous, lifestyle is supported by the presence the bug's completely coriaceous forewings and sclerotized abdominal ventrites that would not allow for large abdominal expansion following a blood meal (Schuh and Slater, 1995). A flexible abdomen is necessary for facilitating hematophagy because in extant bedbugs a single blood meal may represent between 130% and 200% of the body weight of an unfed individual (Reinhardt and Siva-Jothy, 2007; Wattal and Kalra, 1961).

### Defensive or Aggressive Mimicry?

Perhaps the most striking feature of Berstidae is their remarkable resemblance to beetles. They possess a beetle-like body form with coriaceous forewings sclerotized throughout their entire length, longitudinal hemelytral striae, and a pronotum that is anteriorly much wider than the head. In particular, the beetle-like hemelytra are highly atypical of true bugs. While some hemipteran families have a coleopterid body form, the complete sclerotization of the forewings and their parallel arrangement is an unusual feature. This was probably not the result of convergent evolution due to selection by predators such as arachnids (Huang et al., 2018) or ants (Tihelka et al., 2020), as the berstid hemelytral margin is still much smaller than in some myrmecophilous beetles where it confers protection from arthropod predators (Hölldobler and Kwapich, 2017; Olberg, 2015). In addition, this interpretation cannot explain other similarities with beetles such as the presence of subparallel hemelytral veins and the beetle-like head shape, neither of which are directly related to anti-predatory defense.

**Figure 3:**
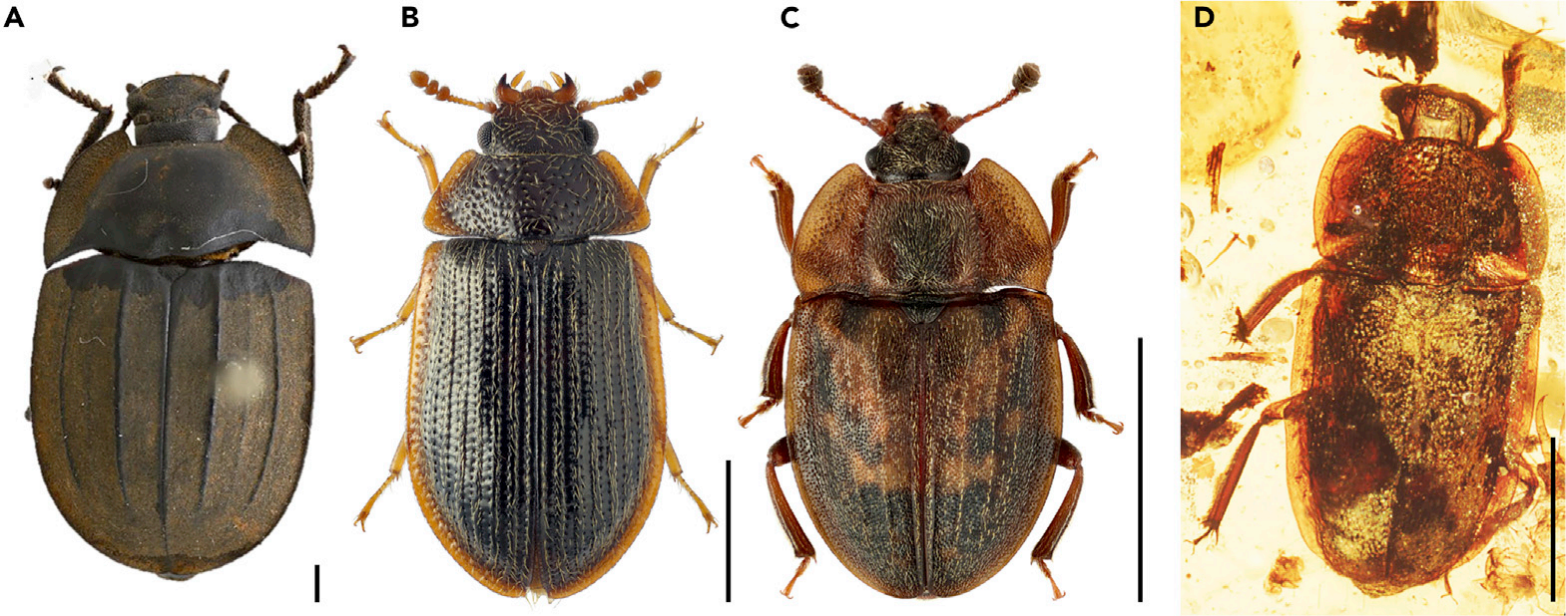
Recent and Fossil Beetles with a Body Plan Similar to Berstidae fam. nov. (A) Recent *Boreosaragus* sp. (Tenebrionidae). Image courtesy of L. Gibson. (B) Recent *Ancyrona japonica* (Trogossitidae). Image courtesy of K.V. Makarov. (C) Recent *Omosita depressa* (Nitidulidae). Image courtesy of M.E. Smirnov. (D) Fossil *Sorodites angustipes* (Nitidulidae) from Burmese amber. Image courtesy of A.G. Kirejtshuk. Scale bars: 1 mm.

In particular, berstids resemble some beetle taxa belonging to the diverse polyphagan families Trogossitidae (bark-gnawing beetles), Nitidulidae (sap beetles), and Tenebrionidae (darkling beetle, namely the extant Australian pie-dish beetles), some of which occur under bark or in dead wood where they feed on fungi, organic remains, or are predaceous (Figure 3). All the three aforementioned families are recorded from or believed to have occurred in the same Cretaceous amber fauna (Kirejtshuk and Chetverikov, 2018; Peris et al., 2014; Ross, 2019). Recently, an interesting sap beetle has been documented from this same amber deposit that exhibits striking superficial similarities to *Bersta* (Kirejtshuk and Chetverikov, 2018). Given the marked similarity between berstids and polyphagan beetles and the likely co-occurrence of the two, it is reasonable to assume that the former mimicked the latter.

But why may have berstids mimicked beetles? There appear to be two plausible explanations: defensive or aggressive mimicry. In the former case, the mimic signals either genuine or false unprofitability to the predator, usually in terms of unpalatability or toxicity. The majority of the beetles that berstids resemble are not known to be aposematic, noxious, or otherwise harmful (Schmidt, 1990), although the existence of toxic or distasteful species in the Cretaceous cannot be ruled out. It seems more probable that the tough exoskeleton of most beetles may make them less palatable than soft-bodied insects to some predators. Some extant true bug families such as Scutelleridae have a remarkably coleopteran-like body plan, although it is not known whether it contributes to defensive mimicry (Schuh and Slater, 1995).

**Figure 4:**
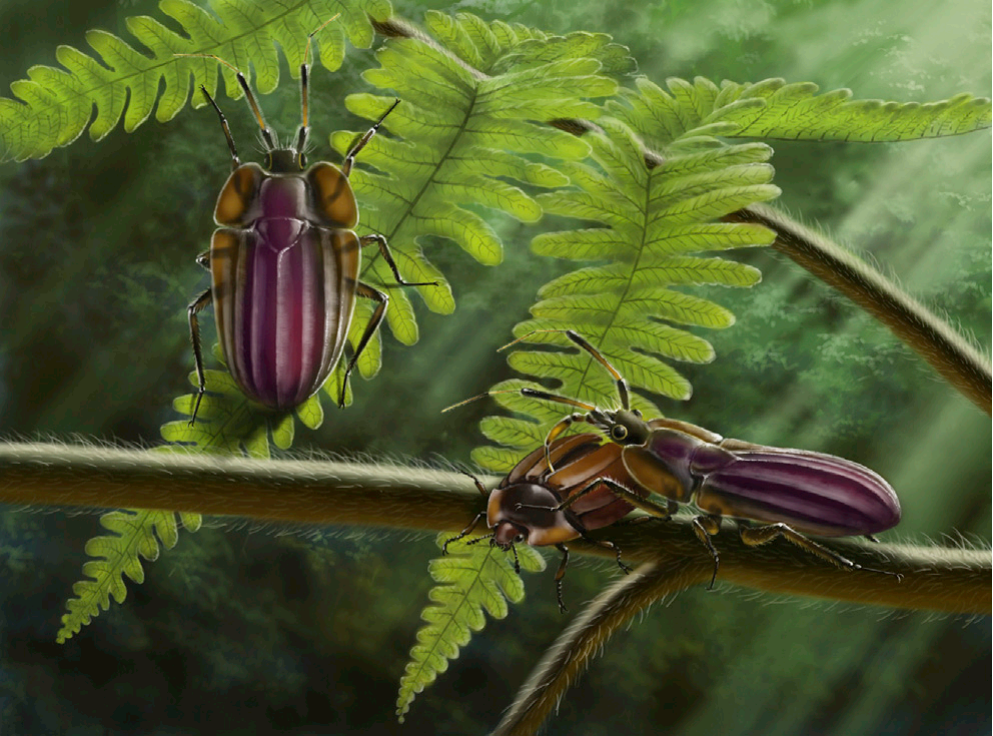
A “Wolf in Sheep's Clothing”? Paleoecological reconstruction of *Bersta vampirica* gen. et sp. nov. showing one scenario of beetle mimicry in Berstidae. The berstid bug, herein depicted as an aggressive mimic, is praying on a sap beetle (Nitidulidae) in the Cretaceous amber forest, ∼99 Ma. Artwork by Mr. Jie Sun.

Aggressive mimicry, whereby a predator tries to “fool” a prey species by looking like the prey itself or another species that is beneficial or harmless to the prey (Ruxton et al., 2019), has been reported from several groups of extant true bugs that feed on ants (Mclver and Stonedahl, 1993) and spiders (Wignall and Taylor, 2011, 2010). By resembling harmless beetles, species of *Bersta* could have approached their prey without eliciting an escape response. Just like some modern predaceous bugs, berstids may have fed on nymphs and adults of small insects (Schuh and Slater, 1995). Given that some bark-gnawing and darkling beetles are predaceous themselves, the bugs may have targeted these model beetles, as other insects would most likely avoid a potential predator (Snyder and Wise, 2000). Aggressive mimicry between bugs and beetles has been hypothesized in at least one extant species; the coloration and gross morphology of the predaceous nymphs *Afrius purpureus* (Pentatomidae) appear remarkably similar to the leaf beetle *Mesoplatys cincta* (Chrysomelidae). Both species frequently co-occur on riverhemp (*Sesbania* spp.) in Senegal, and it has been indicated that the striking similarity may enable the *Afrius* to approach the beetles without eliciting an escape response close enough to allow for capture (Bourdouxhe and Jolivet, 1981). In either way, it is possible that berstid mimicry was neither exclusively defensive nor aggressive, and that the bugs preyed on other species aside from the beetles they mimicked. A hypothetical reconstruction of berstids as aggressive mimics of beetles is depicted in Figure 4.

Although the similarity between berstids and polyphagan beetles is not perfect, many cases of imperfect resemblance between mimics and their models are known (Kikuchi and Pfennig, 2013; Pfennig, 2012). Mimicry is always dependent on the sensory abilities of the organism being deceived, and so, for example, although many hoverflies may be easily distinguished by the trained human eye from the bees and wasps they mimic, they are sufficiently similar to deceive their predators (Kikuchi and Pfennig, 2013; Kraemer and Adams, 2014; Penney et al., 2012). With reference to the presence of metathoracic scent glands in *Bersta*, it is possible that the bugs also employed chemical mimicry to further conceal themselves from their prey. Although admittedly traces of chemicals in amber are difficult to detect (McCoy et al., 2019), it is likely that chemical mimicry did exist in some Cretaceous insects, such as in termitophilous beetles that may have used chemical camouflage to infiltrate termite nests (Cai et al., 2017).

### The Fossil Record of Insect Mimicry

To our knowledge, berstids represent the earliest potential example of aggressive mimicry in the invertebrate fossil record. Mimicry is difficult to recognize even in extant faunas, so it should not be surprising that its detection is even more problematic in the fossil record. Insects with leaf-mimicking wings, a form of camouflage, have existed since at least the Permian (Garrouste et al., 2016; Wang et al., 2012). Among Mesozoic insects, liverwort-mimicking larvae from the Cretaceous mimicked their surroundings to prevent predation, thus qualifying as mimesis or masquerade, as contrasted to true aggressive mimicry in berstids, which involved resemblance between predators and their prey with the aim of deceiving the latter (Liu et al., 2018). Further examples of camouflage are documented from Cretaceous amber lacewings (Liu et al., 2018; Pérez-de la Fuente et al., 2018; Wang et al., 2016) and planthoppers (Jiang et al., 2019), but these cases again involve mimesis of inanimate objects and not resemblance of prey species as is the case in berstids. Moreover, mimesis in these lacewings is based on a constructed concealment rather than morphological modifications (i.e., physical mimicry) of the body as is documented here.

The earliest occurrence of Batesian mimicry has been inferred in alienopterid cockroaches from the Early Cretaceous Crato Formation in Brazil; this group of cockroaches also occurs in Burmese amber (Bai et al., 2016; Hinkelman, 2020; Vršanský et al., 2018). Other cases of defensive mimicry in Burmese amber include a wasp-mimicking zhangsolvid fly (Grimaldi, 2016) and coleopterans possibly mimicking noxious net-winged beetles (Lycidae) (Bocák et al., 2019; Poinar and Fanti, 2016).

Although warning coloration and crypsis have been inferred in Carboniferous insects (Jarzembowski, 2005), no convincing examples of aggressive mimicry are known from Paleozoic arthropods. Only the extinct giant Lower Paleozoic sea scorpions (Eurypterida) have been suggested as possible aggressive mimics in the past. Lamont (1969) hypothesized that the apparently “scaly” cuticle of *Parastylonurus* could have been used to conceal the predator from its fish prey. However, this claim is problematic on several accounts. First, eurypterids are rather large “scorpion-like” creatures that share little with fish in terms of their gross morphology. Second, the scaly cuticular ornament is only present in some parts of the body, whereas the rest was apparently unornamented, and the scales display great variability in different body parts (Waterston, 1979). In the absence of comparative studies of eurypterid and fish scales, in terms of their structure and paleocolour, it is most parsimonious to consider this superficial resemblance as a case of convergence, and consequently the hypothesis is not generally accepted (Kácha and Petr, 1996, 1997).

Despite their extreme rarity, it is reasonable to assume that as the number of known mimetic interactions from localities with exceptional fossil preservation grows, we will be better equipped to address the macroevolutionary implications of this ecological phenomenon. Although studies of mimicry in animals made significant progress for decades with very little input from paleontological lines of evidence (Boucot, 1990), the fossil record can provide important insights into the origin and evolution of this complex behavior. Do mimics suffer a higher extinction rate as predicted by their low population sizes, constantly pruned by frequency-dependent selection (Finkbeiner et al., 2018; Lindström et al., 1997; Pfennig et al., 2001), or do mimics represent chronically rare, low-density specialists that are able to persist in low numbers (Vermeij and Grosberg, 2018)? If yes, how? Mimics that parade energetically costly signals are the least likely to be detected (Johnstone and Grafen, 1993), but as the models learn to recognize their mimics (Cheney and Côté, 2005) these will be forced to invest in even costlier signals. How often must mimics innovate to keep pace with their models and can the models outcompete their mimics by forcing them to invest more than they can afford to lose? The expanding wealth of data from cases of fossil mimicry open up new questions with important implications for behavioral ecology and evolutionary biology that can be answered by combining insights from extant species, fossils, and mathematical modeling.

### Limitations of the Study

The fossil record of mimicry in insects, and invertebrates more generally, is at present far too sparse to be used to test hypotheses about the origin of this behavior. More work will be required before fossils can be used to answer macroevolutionary questions about the emergence of mimicry.

### Resource Availability

#### Lead Contact

Further information and requests for resources should be directed to and will be fulfilled by the Lead Contact, Chenyang Cai (cycai@nigpas.ac.cn).

#### Materials Availability

This study did not generate new unique reagents.

#### Data and Code Availability

Original data have been deposited to Mendeley Data: https://data.mendeley.com/datasets/6vk54mggfg/2 and in the Supplemental Information.

## Methods

All methods can be found in the accompanying Transparent Methods supplemental file.

## Supplemental Information

Document S1. Transparent Methods, Figures S1–S5, and Table S1

## Footnotes

Supplemental Information can be found online at https://doi.org/10.1016/j.isci.2020.101280.
