# Supplementary material for: Mimicry in Cretaceous Bugs
Source: iScience. 2020 Jun 16;23(7):101280. doi: 10.1016/j.isci.2020.101280 (PMC7334408; doi:10.1016/j.isci.2020.101280)
Supplement: Document S1. Transparent Methods, Figures S1–S5, and Table S1 [file mmc1.pdf]

iScience, Volume *23*

Supplemental Information

Mimicry in Cretaceous Bugs

Erik Tihelka, Michael S. Engel, Diying Huang, and Chenyang Cai

Figure S1. Morphological details of *Bersta vampirica* gen. et sp. nov. (NIGP171324, holotype), related to Figure 1.

(A) Dorsal view under reflected light.

(B) Ventral view under reflected light.

(C) Hemelytra in dorsal view under reflected light.

(D) Head and pronotum in dorsal view under green fluorescence.

Scale bars (A, B) 500 μm; (C, D) 200 μm. Abbreviations: 1–3, hemelytral veins 1–3; mscu, mesoscutum; pd, pronotal depression; scu, scutellum; sor, supraocular ridges; sutural margin.

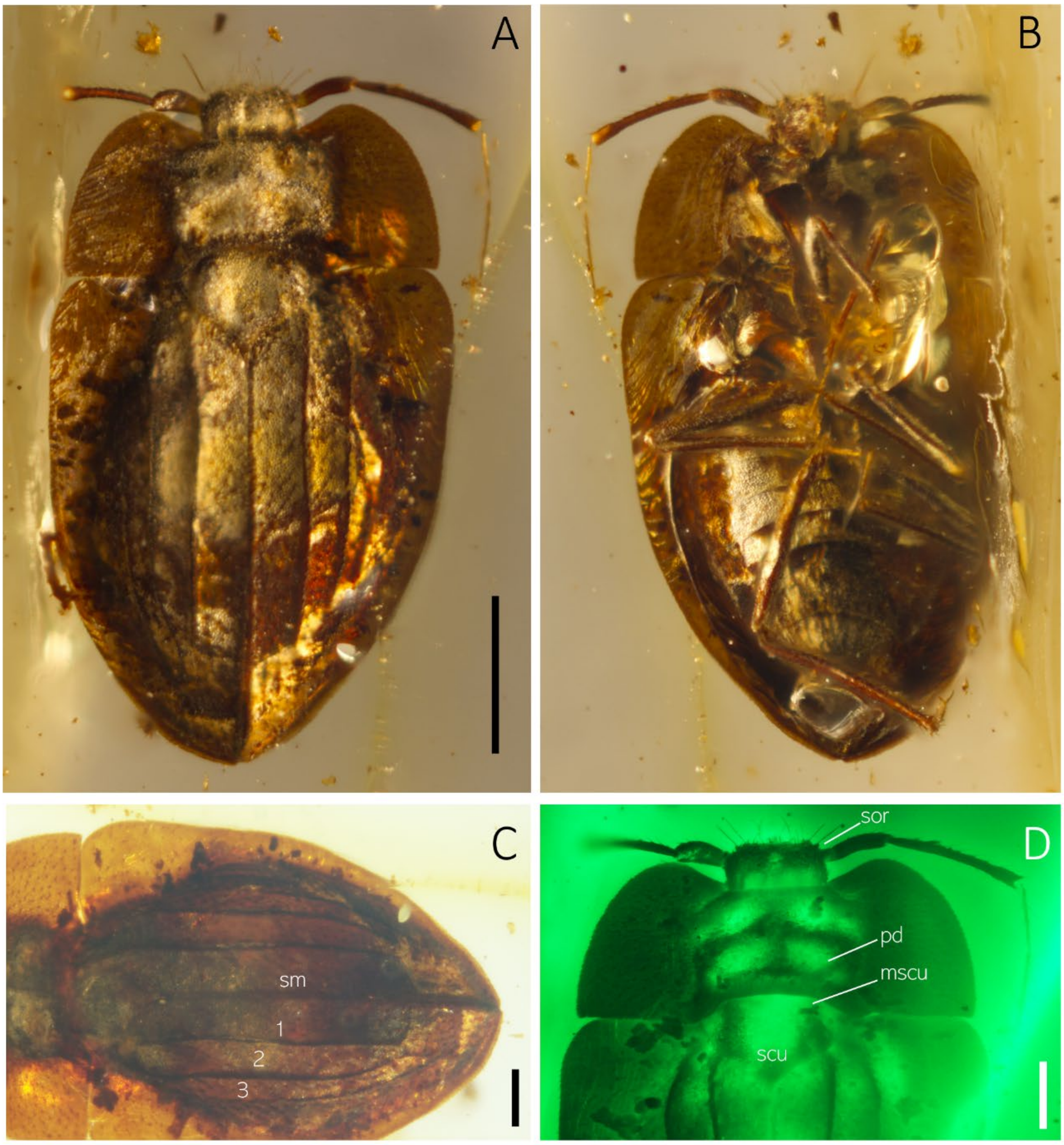

Figure S2. Morphological details of *Bersta vampirica* gen. et sp. nov. (NIGP171324, holotype; NIGP171325, paratype), related to Figure 1.

(A) Dorsal view of paratype under reflected light.

(B) Lateral view of holotype under reflected light.

Scale bars (A, B) 500 μm. Abbreviations: abd, abdomen; cly, clypeus; lb 1–4, labial segments 1–4; mtfm, metafemur.

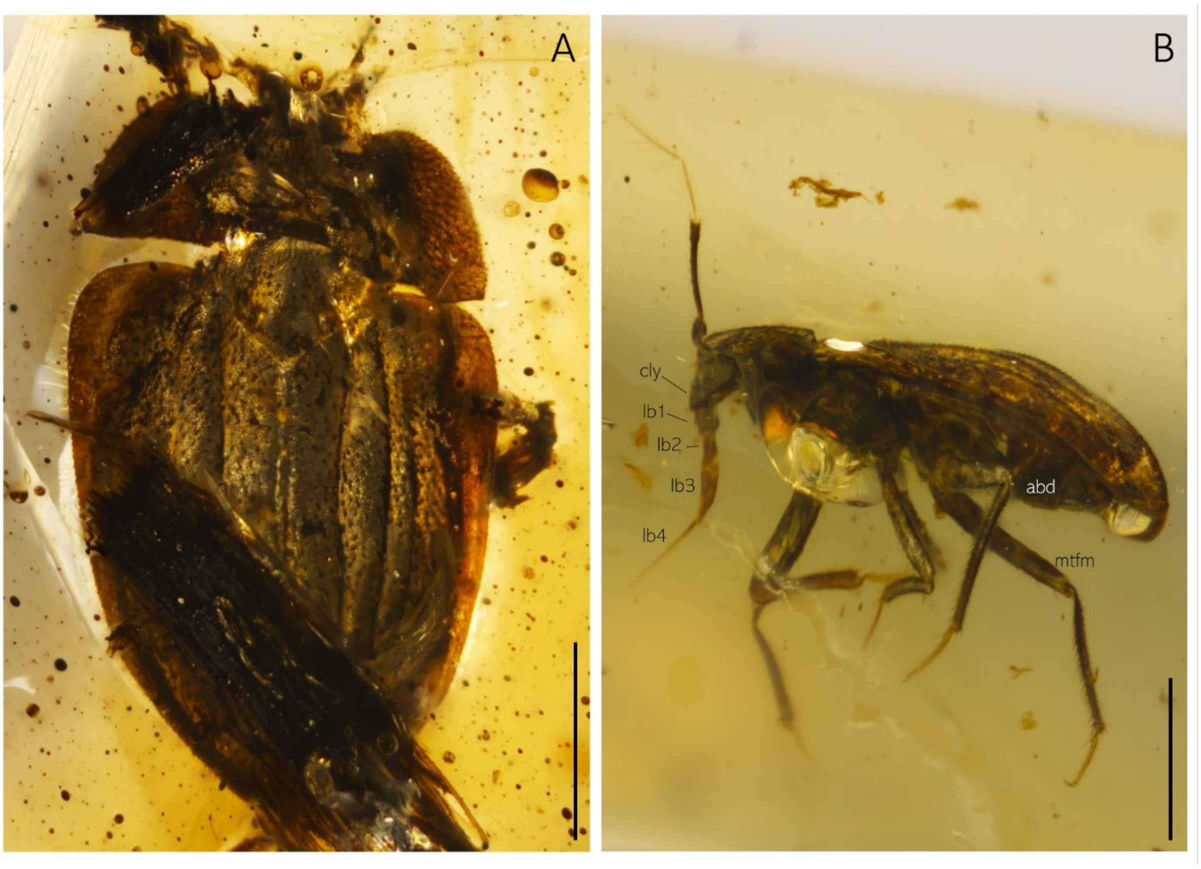

Figure S3. Morphological details of *Bersta coleopteromorpha* gen. et sp. nov. (NIGP171323), related to Figure 1.

(A) Dorsal view under green fluorescence.

(B) Ventral view under reflected light.

(C) Head and pronotum in dorsal view under reflected light.

(D) Prothoracic leg under reflected light.

(E) Hemelytra in dorsal view under reflected light.

Scale bars (A, B, E) 500 μm; (C, D) 200 μm. Abbreviations: 1–3, hemelytral veins 1–3; lar, latitudinal pronotal ridges 1–2; lor, longitudinal pronotal ridges 1 –4; pfm, profemur; prnm, pronotal margin; ptb, protibia; ptbc, protibial cushion; scu, scutellum; sm, sutural margin; sos, supraocular setae.

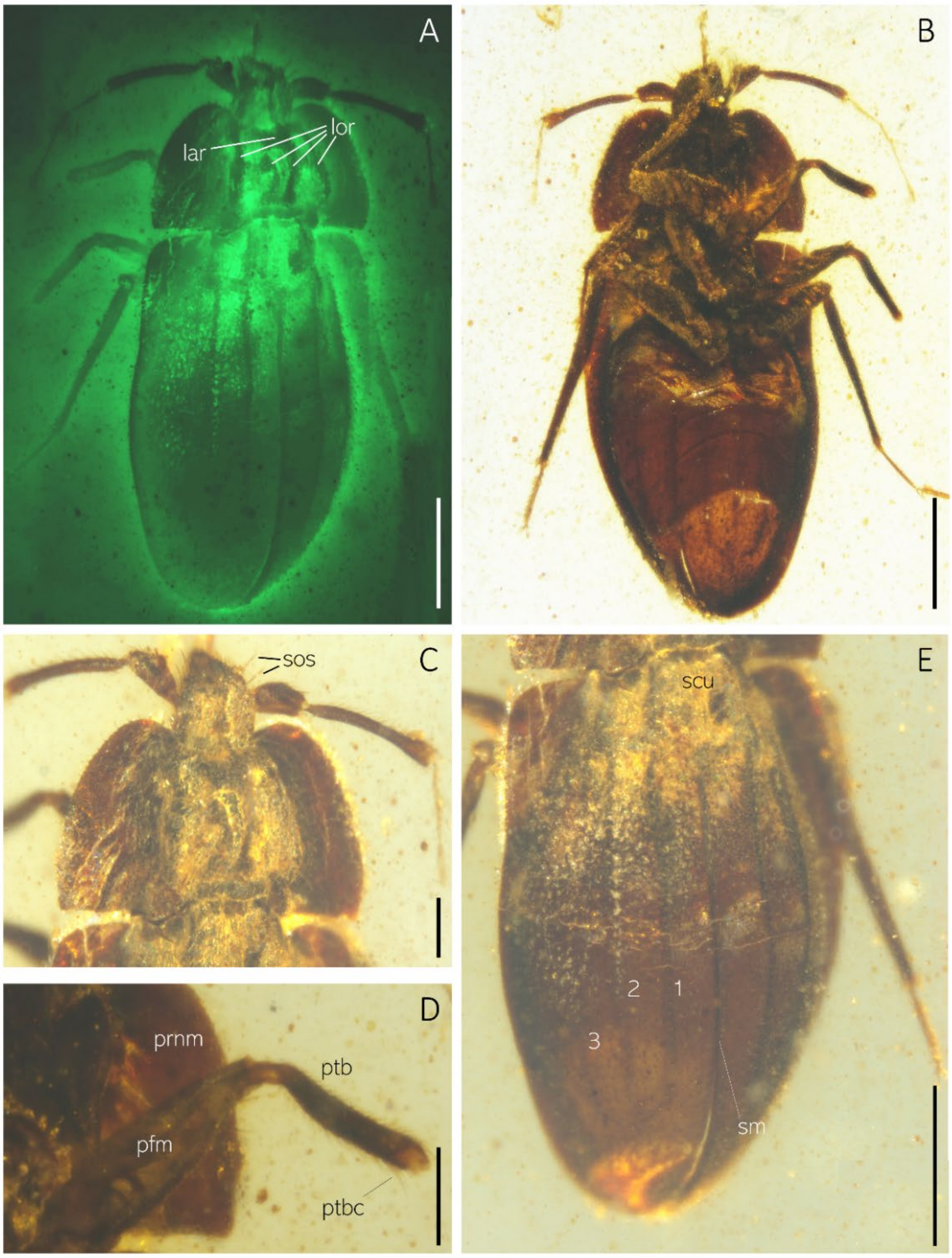

Figure S4. Phylogeny of Cimicomorpha inferred from a CAT-GTR analysis of mitogenomes (P123RNA), related to Figure 2.

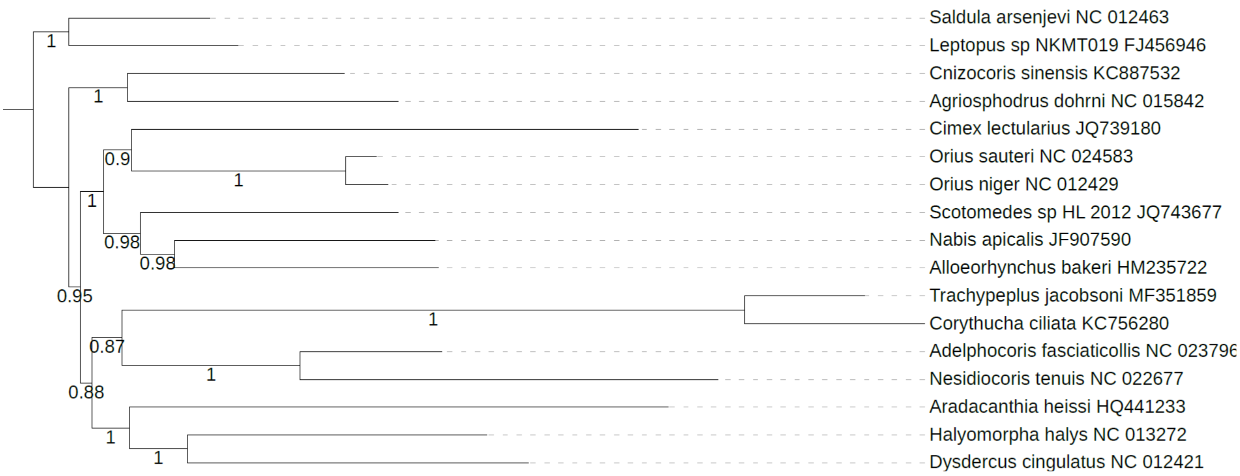

Figure S5. A majority-rule consensus cladogram of four most parsimonious trees (tree length = 297 steps; consistency index = 0.391; retention index = 0.749) showing the systematic position of *Bersta vampirica* gen. et sp. nov., with mapped character states, related to Figure 2. Characters are numbered starting from 0, such as that the first character in the matrix is displayed as character 0.

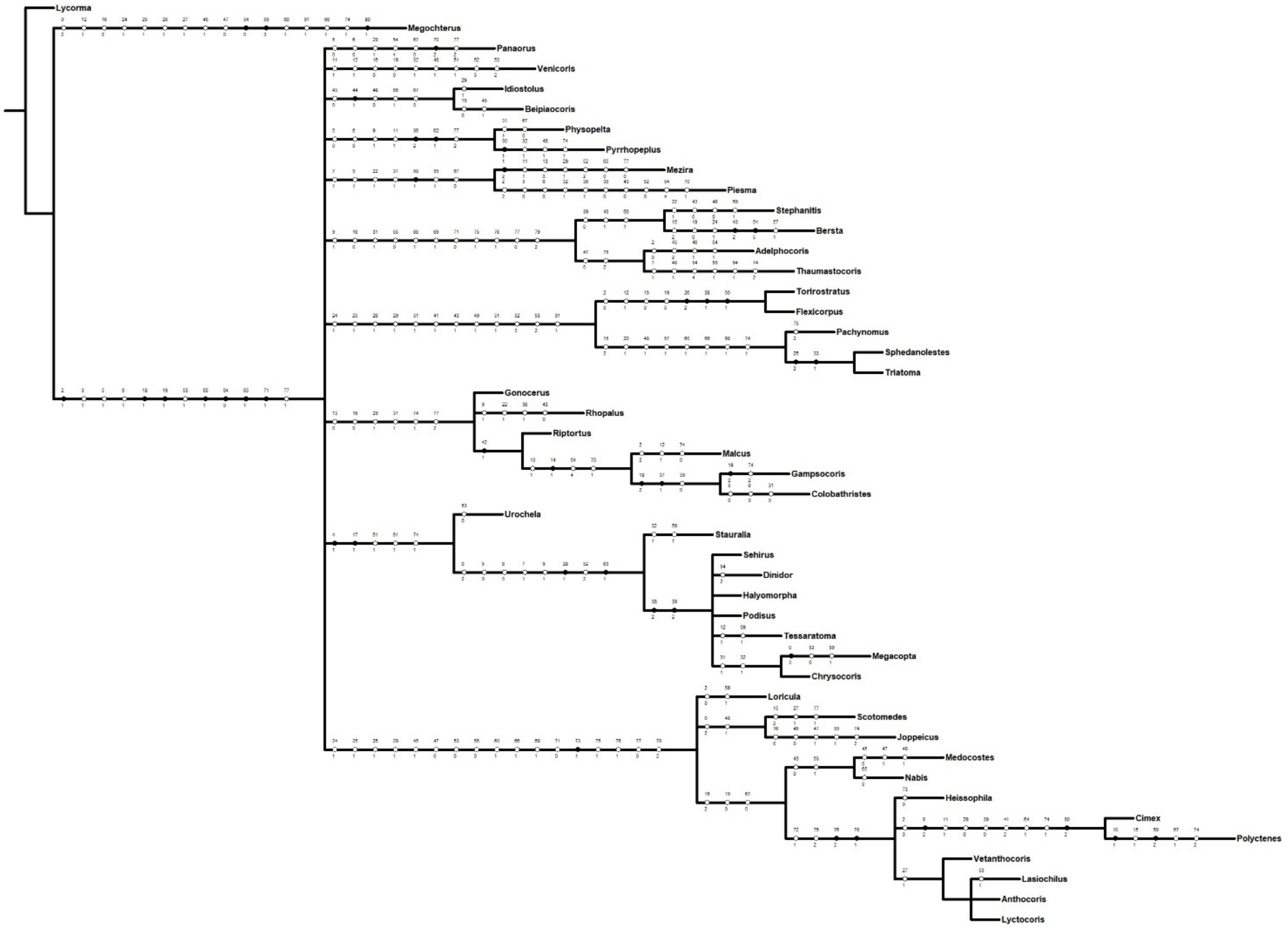

Table S1. GenBank accession numbers for taxa used in the mitogenome analysis, related to Figures 2, S5, and S6.. Systematics follow Schuh and Weirauch (2020).

Taxon	mtDNA
**C I M I C O M O R P H A**	
**REDUVIOIDEA**	
**Reduviidae**	
Agriosphodrus dohrni	NC_015842
Cnizocoris sinensis	KC887532
**NABOIDEA**	
**Nabidae**	
Alloeorhynchus sp.	HM235722
Nabis apicalis	JF907590
**Velocipedidae**	
Scotomedes sp.	JQ743677
**CIMICOIDEA**	
**Anthocoridae**	
Orius sauteri	NC_024583
Orius niger	NC_012429
**Cimicidae**	
Cimex lectularius	JQ739180
**MIROIDEA**	
**Miridae**	
Nesidiocoris tenuis	NC_022677
Adelphocoris suturalis	NC_023796
**Tingidae**	
Corythucha ciliata	KC756280
Trachypeplus jacobsoni	MF351859
**P E N T A T O M O M O R P H A**	
**PENTATOMOIDEA**	
**Pentatomidae**	
Halyomorpha halys	NC_013272
**PYRRHOCOROIDEA**	
**Pyrrhocoridae**	
Dysdercus cingulatus	NC_012421

Transparent methods

Fossil preparation

The amber inclusions originate from a mine at the slope of the Noije Bum hill in the Hukawng Valley, Kachin State, northern Myanmar. The amber piece was polished using sandpapers of gradually finer grits and finally with diatomite mud prior to photography. The type material is deposited in the Nanjing Institute of Geology and Palaeontology, Chinese Academy of Sciences, Nanjing, China. All new taxonomic acts are registered in ZooBank under the publication LSID urn:lsid:zoobank.org:pub:F2D47B4A-0F3A-43AB-814B-3D92A7A9506D.

Photographs under reflected light were taken using a Canon EOS 5D Mark III digital camera, equipped with a Canon MP-E 65 mm macro lens (F2.8, 1–5X), and with an attached Canon MT-24EX twin flash. Fluorescence images with a green background were taken using a Zeiss Axio Imager 2 microscope equipped with a mercury lamp and using the eGFP filter. Helicon Remote 3.9.9 and Helicon Focus 3.10 were used to capture and stack images to increase the depth of field.

Phylogenetic analysis

A total evidence phylogenetic analysis combining both molecular and morphological data was performed to determine the systematic position of Berstidae fam. nov. To reconstruct deep relationships within Cimicomorpha, which have been traditionally difficult to resolve based on morphological data alone, we used published mitochondrial genome sequences (P123RNA for 12 ingroup taxa). To determine the position of Berstidae fam. nov., we used the character matrix of Yao et al. (2014) (81 characters for 46 taxa). We did not use the more recent character matrix of Weirauch et al. (2019), since it uses many characters that are difficult to score in fossil taxa. Taxa belonging to Pentatomorpha, Fulgoromorpha, and Nepomorpha were used as outgroups.

GenBank accessions for the analysed mitogenomes are provided in Table S1. The mitochondrial genomes were downloaded from GenBank using PhyloSuite v 1.2.1 (Zhang et al., 2020). Protein-encoding genes were aligned using the G-INS-i algorithm implemented in the MAFFT v 7.313 plugin within PhyloSuite (Katoh and Standley, 2013), while the ribosomal RNAs were aligned using the E-INS-I algorithm, and the aligned genes were concatenated using PhyloSuite. Concatenation was carried out in PhyloSuite. The decisive matrix was analysed using the site-heterogeneous CAT-GTR+G model in PhyloBayes MPI 1.7 (Lartillot et al., 2013); two independent Markov chain Monte Carlo (MCMC) chains were run until convergence (maxdiff < 0.3).

We tried to separately analyze data for four genes (16S, 18S, 28S, and COI) to shed light on the systematic position of more cimicomorph families, but the recovered tree was poorly supported. Evidently, a small four gene dataset is not sufficient for resolving family-level relationships within Cimicomorpha.

For the morphological data, maximum parsimony analyses were performed in TNT v 1.5 (Goloboff and Catalano, 2016) using the New Technology Search and implied weighting. The recommended value of k = 12 was used (Goloboff et al., 2018). Collapsing rules were set to 'none'. The analysis was run using default settings in 'New Technology Search'. A majority-rule consensus tree was calculated, and a nonparametric bootstrap analysis run with 1,000 replicates. Character states were mapped using ASADO v. 1.61 (Nixon, 2004). The original dataset provided by Yao et al. (2014) was used, which was designed specifically for testing the systematic position of extinct families. In total, 50 out of the total 81 available characters could be scored for *B. vampirica* gen. et sp. nov. The following three new character states were introduced to describe morphological structures observed in Berstidae fam. nov. but not present in the other taxa: character 41 (2: forewings held flat over the abdomen, adjacent to each other, coriaceous throughout their length), character 42 (3: forewing completely coriaceous) and character 55 (5: hemelytral membrane not present). All characters were unordered and equally weighted. All characters were unordered and equally weighted.

The two phylogenetic analyses were run separately, and their results are displayed in Figs. S5–6. A phylogenetic hypothesis on the placement of Berstidae, using mitogenome data to inform deeper nodes, morphological data to infer shallower nodes, and enforcing the monophyly of Miroidea strongly supported by molecular analyses after accounting for compositional heterogeneity (Yang et

al., 2018), is presented in Fig. 3. Datasets, analysis files, and output files are available at Mendeley Data https://data.mendeley.com/datasets/6vk54mggfg/2 (DOI: 10.17632/6vk54mggfg.2).

Supplemental description and taxonomic treatment

Berstidae fam. nov.

Type genus. *Bersta* gen. nov.

Diagnosis. Body elongate to subelliptical, glabrous, not covered with layers of setae, coleopteroid. Size rather small, when compared to the remainder of Hemiptera, length ranging from 2.0 to 2.6 mm. Head hypognathous, more or less pentagonal in dorsal view, without a constriction posterior to compound eyes, collar present. Labium 4-segmented, tapering apically, inserted on ventral head surface. Antenna with four articles; prepedicellite absent; antennomere II (pedicel,) longer than antennomere III (basiflagellomere), antennomeres III and IV (distiflagellomere) filiform, much narrower than preceding antennomeres, with erect setae. Compound eyes well-developed, but not surpassing collar posteriorly. Ocelli absent. Thoracic labial groove present; pronotum trapezoidal. Pronotum almost butterfly-shaped, with anterior margin sinuate, anterior angles smoothly curved, and posterior angles approximately right-angled. Pronotal and hemelytral margins pronouncedly expanded, clearly overlapping sides of thorax and abdominal base. Legs slender and setose, of cursorial type. Prolegs lack any specialist raptorial adaptations apart from the presence of a minute setose cushion-like structure at the apex. Tarsi trimerous, pretarsal parempodia absent.

Berstids differ from other hemipteran families with a coleopteroid appearance, such as Omaniidae, Schizopteridae, and Tingidae, in having their forewings coriaceous, sclerotized throughout their posterior margins, parallel, and connate (apices not overlapping), each with simple longitudinal veins, and lacking a costal fracture. They can be assigned to Cimicomorpha based on the presence of cephalic trichobothria, and setose cushion on the forelegs, body not covered with a short hair pile and lacking marginal laminae; head not transversely constricted or divided into two distinct lobes; eyes small; antennae not concealed below head in grooves under compound eyes, longer than head, antennomere 2 longer than antennomere 1; forewings modified into coleopteroid hemelytra with longitudinal carinae; abdominal sterna without trichobothria placed sublaterally or submedially; protibiae not flattened; tarsi 3-segmented, claws lacking pulvilli. Within Cimicomorpha, the fully coriaceous, parallel and non-overlapping hemelytra represent an apomorphy of Berstidae.

Included taxa. A single genus, *Bersta* gen. nov., containing two species.

Bersta gen. nov.

Type species. Bersta vampirica sp. nov.

Diagnosis. As for the family with additional characters: abdominal trichobothria absent, abdominal spiracles on unified sternal plates, each hemelytron with three longitudinal veins, body length ≤ 2.6 mm.

Etymology. The new generic name is a euphonious combination of letters inspired by “Berstuk” a deity of the Wendic Slavs and Sorbs that, according to myth, inhabited deep forests and had the ability to morph into different animals, and refers to the likely paleoenvironment of the bugs and their mimetic association with beetles. The name is considered of feminine gender.

Included species. Two new species: *Bersta vampirica* sp. nov. and *B. coleopteromorpha* sp. nov.

Description. Body elongate to subelliptical, dorsal surface somewhat convex, more or less glabrous. Body length 2.0 to 2.6 mm from clypeus to hemelytral apices, body width 1.0 to 1.2 mm at widest point at the base of the hemelytra. Colour light to dark brown, darker towards the midline, sometimes with a reddish to orange tinge on the pronotal and hemelytral margins.

Head hypognathous, more or less pentagonal in dorsal view, not forming a distinct neck. Clypeus sparsely setate and strongly declivent from the base of the antennae. Mandibular plates short, not reaching to the clypeal apex. Bucculae long, extending over two thirds of the head length. Labium

inserted distally on the head, four-segmented, tapering apically, and reaching approximately to the metacoxae. Compound eyes finely faceted and oval, not protruding, and not visible in dorsal view. Ocelli apparently absent. Three pairs of thick and elongate cephalic macrosetae present, each on a raised carina. Antennae inserted anteroventrally to the compound eyes, antenniferous tubercles absent. Antennae 4-segmented, reaching to the base of forewings. Antennal segment 1 (scape) broad and barrel-shaped, longer than half of head width, sparsely setate towards the apex. Antennal segment 2 (pedicel) 1.8 – 2.0 times longer than scape and narrower, slightly expanding apically, with rows of setae. Preflagelloid present, spherical. Antennal segment 3 (basiflagellum) shorter and distinctly thinner than the preceding two segments, transparent, with rows of erect setae. Antennal segment 4 (distiflagellum) also narrow and transparent, with rows of setae, 1.6 – 1.8 times longer than the preceding segment.

Pronotum 0.36 – 0.46 times as long as wide, widest in its second third, slightly narrower basally, and gradually curving inwards anteriorly, forming a distinct collar such that the anterior apices project to the basal quarter of the head. Posterior angles approximately right-angled, directed posteriorly, not overlapping hemelytral apices. Pronotal surface with sparse, equally spaced, dark setae. Pronotal disc with a raised medial and various lateral ridges. Pronotal margin expanded, up to 2.3 times wider than thorax. Labial groove accommodating no more than two thirds of the labium length. Metathoracic scent glands diastomian. Mesoscutum narrowly exposed. Scutellum 0.27 – 0.28 mm long, pentagonal, depressed medially, with a pointed apex. Scutellum small, representing approximately 1/5 of hemelytron length.

Hemelytra elongate, 1.3 – 1.6 times longer than wide, broadest in the basal quarter and tapering in the posterior half, flattened anteriorly and concave in the apical half. Hemelytra sclerotized throughout their length, membranous apical region absent. Hemelytral apices not overlapping. Each coriaceous hemelytron with three longitudinal raised veins. Vein 1 starting at the scutellum, almost straight. Veins 2 and 3 starting in the basal ninth of the hemelytron, bending medially in the posterior third. Veins never reaching the hemelytral apex, all terminating in the apical ninth of the hemelytra. Distances between veins decreasing laterally. Sutural margin raised or not. Cuneus and costal fracture absent. Hindwings not visible.

Legs slender, with rows of setae. Procoxal lamellae triangular, widest anteriorly and tapering posteriorly, forming a labial groove. Mesocoxae directed posteriorly, metacoxae directed laterally. Profemur clavate, as long as protibia, without spines. Mesofemur obclavate, not shorter than mesotarsi. Metafemur slightly expanded distally, approximately half as long as mesotibia. Tibiae terminating with groups of large thick setae or spines and/or tuft of setae. Tarsi elongate, slender, 3-segmented. Claws simple, gradually curving and tapering towards the apex. Pulvilli absent.

Abdomen broadest medially. Tergites apparently sclerotized, each with spiracles located laterally, borders between tergites most apparent medially. No pores or scars of abdominal glands visible. Ventral laterotergites not visible.

Bersta coleopteromorpha sp. nov.
Figs. 1, S3

Etymology. Derived from 'Coleoptera' (beetle) and the Greek '-morphus' in reference to the species' striking beetle-like appearance.

Diagnosis. Distinguished from *B. vampirica* gen. et sp. nov. by hemelytral vein 1 not connected to the sutural margin and veins 2 and 3 fading posteriorly, not joining, with the posterior part of vein 1 directed laterally and the posterior part of vein 3 directed medially. Moreover, the pronotum has four clearly defined longitudinal ridges connected an anterior latitudinal ridge. Unlike its sister species, *B. B. coleopteromorpha* lacks elevated keels to the side of eyes.

Holotype. NIGP171323, sex undetermined.

Type locality and horizon. Amber mine in the Hukawng Valley, Myitkyina District, Kachin State, Myanmar; Albian/Cenomanian boundary to late Albian (mid-Cretaceous).

Description. Body length 2.52 mm, body width 1.09 mm, 2.3 times as long as wide. Head, pronotal disc, hemelytra along midline, and appendages black to dark brown; two apical antennomeres transparent; rest of body brown.

Head 1.1 times as long as wide. Supraocular setae not positioned on ridges. Ratio of antennal segment lengths (in mm): 0.19 : 0.39 : 0.18 : 0.35. Antennal segment 1 representing 0.7 times of head width. Labium not clearly visible.

Pronotum 0.46 mm long along the medial line, 0.99 mm wide across the broadest point, 3.9 times wider than head. Anterior margin incurved medially, anterior angles not reaching to the posterior part of the antennal insertions. Pronotal disc with three medial longitudinal ridges, the middle one being the shortest and the lateral two connected to each by one latitudinal ridge. A fourth additional lateral ridge is present on the right side and is connected to the complex by an anterior latitudinal ridge. Scutellum with distinctly raised lateral ridges.

Hemelytra 1.73 mm long along medial suture, 1.10 mm wide across the broadest point. Vein 1 distinctly raised, straight throughout its length, gradually fainting posteriorly, with posterior part slightly curved laterally, not fused to the sutural margin. Vein 2 raised and more or less straight, gradually fainting posteriorly, shortest of the three veins. Vein 3 more or less straight, gradually fainting posteriorly, with posterior part slightly curved medially. All veins terminating in the posterior ninth of the hemelytron. Sutural margin raised.

Pro- and mesotibia lacking spines. Protibial apex with a cushion of hairs located ventrally. Protibia 1.4 times longer than protarsi. Mesofemur 1.3 times longer than mesotibia. Mesotibia 1.2 times longer than mesotarsi. Metafemur 1.4 times longer than metatibia. Metatibia with four apical spines arranged in two rows. Metatibia 2.9 times longer than metatarsi.

Abdominal tergites subequal, two abdominal tergites missing, revealing a part of the hollow body cavity, hindwings not visible.

Bersta vampirica sp. nov.
Figs. 1, S1, S2

Etymology. Derived from the Slavic '*vampir*', in reference to the species' likely predaceous habits and its piercing and sucking mouthparts.

Diagnosis. The species can be differentiated from *B. coleopteromorpha* gen. et sp. nov. by the hemelytral vein 1 abruptly curving medially in its posterior part and joining the sutural margin, and veins 2 and 3 fused posteriorly. Moreover, it differs from by the anterior pronotal angles reaching to the posterior part of the antennal insertions, and by pronotum with an indistinct longitudinal and latitudinal ridge forming a raised cross-like structure with four depressions. Additionally, it can be distinguished from *B. coleopteromorpha* gen. et sp. nov. by the presence of supraocular ridges, each with a single seta.

Holotype. NIGP171324, sex undetermined.

Paratype. NIGP171325, sex undetermined.

Type locality and horizon. Amber mine in the Hukawng Valley, Myitkyina District, Kachin State, Myanmar; Albian/Cenomanian boundary to late Albian (mid-Cretaceous).

Description. Body length 2.00 mm (2.37 mm in paratype), body width 1.20 mm (1.34 mm in paratype), 1.7 times as long as wide (1.8 times in paratype). Head, pronotal disc, hemelytra along midline, and appendages black to dark brown; two apical antennomeres transparent; expanded pronotal and hemelytral margins distinctly bright brown.

Head 1.1 times as long as wide. Supraocular setae positioned on short ridges. Ratio of antennal segment lengths (in mm): 0.20 : 0.37 : 0.19 : 0.34. Antennal segment 1 representing 0.8 times of head width. Labial segment 1 stout and almost as long as wide, labial segment 2 slightly longer and equally

wide throughout, labial segment 3 tapering in the distal quarter, labial segment 4 tapering throughout its length and subequal to the preceding segment.

Pronotum 0.38 mm long along the medial line (0.37 in paratype), 1.06 mm wide across the broadest point (1.26 mm in paratype), 3.8 times wider than head (4.1 in paratype). Anterior margin strongly incurved medially, anterior angles reaching to the posterior parts of the antennal insertions. Pronotal disc with indistinctly raised longitudinal and latitudinal ridges intersecting and forming a cross-like structure surrounded by four oval depressions. Scutellum slightly raised laterally.

Hemelytra 1.50 mm long along medial suture (1.74 mm in paratype), 1.16 mm wide across the broadest point (1.34 mm in paratype). Vein 1 distinctly raised, abruptly curving medially in its posteriormost part and joining the sutural margin. Vein 2 raised, curved in the posterior third, straight in the posterior ninth, fusing with vein 2. Vein 3 indistinct, not easily visible in its basal half, curved medially in its posterior third. Sutural margin raised.

Tibiae terminating with a tuft of large setae or spines. Protibial apex with a patch of setae. Protibia 1.8 times longer than protarsi. Mesofemur 1.2 times longer than mesotibia. Mesotibia 2.4 times longer than mesotarsi. Metafemur as long as metatibia.

Abdominal tergites subequal, two apical tergites each approximately twice the width of the preceding segments.

Systematic position

The present fossils can be placed into the infraorder Cimicomorpha on the basis of the combination of the following characters: body not covered with a short hair pile and lacking marginal laminae; head not transversely constricted or divided into two distinct lobes, with trichobothria; eyes small; antennae not concealed below head in grooves under compound eyes, longer than head, antennomere 2 longer than antennomere 1; forewings modified into coleopteroid hemelytra with longitudinal carinae; abdominal sterna without trichobothria placed sublaterally or submedially; protibiae not flattened; tarsi 3-segmented, claws lacking pulvilli (Schuh and Slater, 1995). Moreover, the foretibia of *B. coleopteromorpha* gen. et sp. nov. and *B. vampirica* gen. et sp. nov. possess small cushion-like structures, variably preserved in our specimens. These cushion-like structure preserved ventrally at the apex of the tibiae are structurally dissimilar to the fossula spongiosa traditionally considered to be unique to Cimicomorpha (Kerzhner, 1981). The fossula spongiosa is in fact apparently absent in the extinct family Vetanthocoridae (Tang et al., 2017; Yao et al., 2014) and phylogenetic studies suggest that it likely emerged independently multiple times in predatory and hematophagous cimicomorphs (Schuh et al., 2009).

To ascertain the systematic position of the beetle-mimicking bugs, a phylogenetic analysis was performed. The morphological analyses resulted into four most parsimonious trees. The majority consensus tree (tree length = 297 steps; consistency index = 0.391; retention index = 0.749) recovered *B. vampirica* gen. et sp. nov. as a sister group to the lace bug genus *Stephanitis* (Tingidae) (Fig. 3, S4). Berstidae fam. nov. is united with the recent Tingidae by the presence of long and narrow bucculae, absence of armature on the scutellum, and corium size. Indeed, some modern members of Tingidae share with *Bersta* gen. nov. the presence of highly sclerotized forewings with raised veins (e.g. genus *Physatocheila*), giving them a highly superficial beetle-like appearance. The new family Berstidae can be easily distinguished from all other cimicomorph families including Tingidae by the hemelytron thickened throughout its length and lacking a membranous apical region. The loss of the costal fracture is reminiscent of some Joppeicidae and taxa with staphylinoid hemelytra (Schuh et al., 2009). Berstidae fam. nov. can also be distinguished from all other cimicomorph families by the apparent absence of parempodia (Schuh et al., 2009). It is also notable that *Bersta* has a longer scape than other fossils such as *Beipiaocoris* that have been sampled in the study. Another unusual characteristic of the beetle-mimicking bugs is their small size, they are significantly smaller than most heteropterans; the smallest adult hemipterans reach around 2 mm in length (Schuh et al., 2008). The new family can also be easily distinguished from all fossil taxa; Berstids are also the only Mesozoic true bugs without a trapezoidal pronotum (Yao et al., 2014).

Supplemental references

Goloboff, P.A., Catalano, S.A., 2016. TNT version 1.5, including a full implementation of phylogenetic morphometrics. Cladistics 32, 221–238. https://doi.org/10.1111/cla.12160

Goloboff, P.A., Torres, A., Arias, J.S., 2018. Weighted parsimony outperforms other methods of phylogenetic inference under models appropriate for morphology. Cladistics 34, 407–437. https://doi.org/10.1111/cla.12205

Haridass, E.T., Ananthakrishnan, T.N., 1980. Functional morphology of the fossula spongiosa in some reduviids (Insecta—Heteroptera—Reduviidae). Proc. Ind. Acad. Sci. Anim. Sci. 89, 457–466.

Katoh, K., Standley, D.M., 2013. MAFFT Multiple Sequence Alignment Software Version 7: Improvements in performance and usability. Mol. Biol. Evol. 30, 772–780. https://doi.org/10.1093/molbev/mst010

Kerzhner, I.M., 1981. Fauna of the USSR. Bugs. Volume 13, No. 2. Heteroptera of the Family Nabidae. Nauka, Leningrad.

Lartillot, N., Rodrigue, N., Stubbs, D., Richer, J., 2013. PhyloBayes MPI: Phylogenetic reconstruction with infinite mixtures of profiles in a parallel environment. Syst. Biol. 62, 611–615. https://doi.org/10.1093/sysbio/syt022

Nixon, K.C., 2004. ASADO version 1.5 Beta. Program and Documentation Distributed by the Author. Self-published, Ithaca.

Schuh, R.T., Slater, J.A., 1995. True bugs of the world (Hemiptera: Heteroptera): Classification and natural history. Cornell Univeristy Press, Ithaca.

Schuh, R.T., Weirauch, C., 2020. True Bugs of the World (Hemiptera: Heteroptera), 2nd ed. Siri Scientific Press, Manchester.

Schuh, R.T., Weirauch, C., Henry, T.J., Halbert, S.E., 2008. Curaliidae, a New Family of Heteroptera (Insecta: Hemiptera) from the Eastern United States. Ann Entomol Soc Am 101, 20–29. https://doi.org/10.1603/0013-8746(2008)101[20:CANFOH]2.0.CO;2

Schuh, R.T., Weirauch, C., Wheeler, W.C., 2009. Phylogenetic relationships within the Cimicomorpha (Hemiptera: Heteroptera): a total-evidence analysis. Systematic Entomology 34, 15–48. https://doi.org/10.1111/j.1365-3113.2008.00436.x

Tang, D., Yao, Y., Ren, D., 2017. Phylogenetic position of the extinct insect family Vetanthocoridae (Heteroptera) in Cimiciformes. Journal of Systematic Palaeontology 15, 697–708. https://doi.org/10.1080/14772019.2016.1219779

Weirauch, C., Schuh, R.T., Cassis, G., Wheeler, W.C., 2019. Revisiting habitat and lifestyle transitions in Heteroptera (Insecta: Hemiptera): insights from a combined morphological and molecular phylogeny. Cladistics 35, 67–105. https://doi.org/10.1111/cla.12233

Yang, H., Li, T., Dang, K., Bu, W., 2018. Compositional and mutational rate heterogeneity in mitochondrial genomes and its effect on the phylogenetic inferences of Cimicomorpha (Hemiptera: Heteroptera). BMC Genomics 19, 264. https://doi.org/10.1186/s12864-018-4650-9

Yao, Y., Cai, W., Xu, X., Shih, C., Engel, M.S., Zheng, X., Zhao, Y., Ren, D., 2014. Blood-Feeding True Bugs in the Early Cretaceous. Current Biology 24, 1786–1792. https://doi.org/10.1016/j.cub.2014.06.045

Zhang, D., Gao, F., Jakovlić, I., Zou, H., Zhang, J., Li, W.X., Wang, G.T., 2020. PhyloSuite: An integrated and scalable desktop platform for streamlined molecular sequence data management and evolutionary phylogenetics studies. Mol. Ecol. Res. 20, 348–355. https://doi.org/10.1111/1755-0998.13096
